# Fever, Myositis, and Paralysis: Is This Inflammatory Myopathy or Neuroinvasive Disease?

**DOI:** 10.1155/2016/5395249

**Published:** 2016-03-28

**Authors:** Aneeta R. Kiran, Richard A. Lau, Kim M. Wu, Andrew L. Wong, Philip J. Clements, Emil R. Heinze

**Affiliations:** ^1^UCLA-Olive View Rheumatology Program, Division of Rheumatology, Olive View-UCLA Medical Center, 14445 Olive View Drive, No. 2B182, Sylmar, CA 91342, USA; ^2^UCLA-Olive View Internal Medicine Program, Department of Medicine, Olive View-UCLA Medical Center, 14445 Olive View Drive, No. 2B182, Sylmar, CA 91342, USA

## Abstract

West Nile virus (WNV) is a mosquito-borne RNA* Flavivirus* which emerged in North America in 1999. Most patients present with a febrile illness but a few develop WNV neuroinvasive disease. Myopathy is an uncommon manifestation. We describe a case of a 42-year-old male from Los Angeles who presented with 8 days of fever and muscle pain. Initial physical exam was normal except for 4/5 muscle strength testing in his extremity proximal muscles. Laboratory revealed a creatine kinase of 45,000 and a urinalysis with large blood but no red blood cells, suggesting rhabdomyolysis. The patient's condition declined despite aggressive supportive care and hydration, and on hospital day #6 he developed severe altered mental status and progressed to complete right arm paralysis and 2/5 muscle strength in bilateral legs. EMG/NCS showed sensorimotor axonal polyneuropathy and the cerebrospinal fluid was positive for IgM and IgG WNV antibodies. The patient was diagnosed with WNV neuroinvasive disease, poliomyelitis (and encephalitis) type with myopathy/muscle involvement. He was treated supportively and his muscle and neurologic disease gradually improved. At 12-month follow-up his muscle enzymes had normalized and his weakness had improved to 5/5 strength in bilateral legs and 3/5 strength in the right arm.

## 1. Introduction

West Nile Virus (WNV) is an important arthropod-borne human pathogen which is a neurotropic RNA virus of the Flaviviridae family. Most infections are clinically silent or present as a mild febrile illness. However, more severe neuroinvasive disease can occur resulting in meningitis, encephalitis, or acute anterior poliomyelitis [1, 2]. Myopathy is an uncommon presentation and can occur with creatine kinase levels as high as 45,000 U/L suggestive of rhabdomyolysis [3]. We present a case of WNV neuroinvasive disease complicated by sensorineural axonopathy and severe myopathy presenting as progressive paralysis and rhabdomyolysis.

## 2. Case

A 62-year-old Hispanic male with a past medical history of diabetes, hyperlipidemia, coronary artery disease, and ischemic cardiomyopathy was in his usual state of health until he presented to the emergency room with an eight-day history of fever, rigors, fatigue, whole body muscle pain, and productive cough with yellow sputum. He had decreased appetite and decreased oral intake for seven days. He reported nausea, abdominal discomfort, dark urine, and decreased urinary output for two to three days. On physical exam he was alert and oriented ×4, febrile, and tachycardic and had decreased proximal muscle strength in all his extremities (4/5 in bilateral upper extremities and 3/5 strength in the bilateral lower extremities). The remainder of the physical exam was within normal limits. There was no distal muscle weakness and no sensory deficits. The initial workup included laboratory evaluation which demonstrated WBC of 16.2 thou cells/*μ*L, creatine kinase (CK) of 45,100 U/L, aldolase of 136.8 U/L, elevated aspartate aminotransferase (AST) of 2396 U/L, and alanine transferase (ALT) of 571 U/L, and urinalysis also revealed large blood but no RBCs, all of which was consistent with rhabdomyolysis. The patient was also found to have a troponin leak, with a peak of 2.617 ng/mL, with subsequent transthoracic echocardiogram demonstrating improved ejection fraction of 42% compared to 35% five months priorly and adenosine Myoview stress test showing stable anteroseptal wall defect consistent with previous known myocardial infarction.

The patient was admitted to a monitored bed for management of rhabdomyolysis, likely secondary to statin versus viral induced myopathy, with consideration for autoimmune myopathy as well. Empiric therapy was started for rhabdomyolysis, including statin discontinuation and IV fluid hydration. The patient was also treated with broad spectrum antibiotics until preliminary cultures returned negative, as the patient initially met SIRS criteria at presentation with fevers, tachycardia, and leukocytosis. In regard to the troponin elevation, cardiology consultants treated the patient for a non-ST elevation myocardial infarction secondary to demand ischemia from acute illness with medical optimization of his cardiac medications. The patient responded well to initial medical management, with the creatine kinase trending down after it remained elevated >45,100 U/L during the first four days of admission. The patient's serum creatinine also began trending down after peaking at 4.03 mg/dL, and his troponin trended down to normal after peaking at 2.617 ng/mL. On hospital day #6, however, the patient developed complications of acute mental status change, as well as worsening weakness. On exam, he was noted to be alert and oriented to person only, minimally following commands and with new onset of complete paralysis in the right upper extremity and marked weakness in bilateral lower extremities (2/5 strength). He also had decreased sensation to light touch in bilateral legs and in the right arm. Right biceps and bilateral ankle reflexes were absent. Cranial nerves and fundoscopic exam were normal.

Given the new neurological deficits on top of the existing rhabdomyolysis and nonspecific constitutional symptomology that the patient initially presented with, the differential diagnosis was expanded with the assistance of neurology consultation. Specifically, there was strong concern for possible invasive CNS infections leading to encephalitis, such as HIV, WNV, CMV, EBV, HSV, VZV, HTLV1, Lyme, toxoplasmosis, cysticercosis, mycoplasma, and syphilis, with higher suspicion of viral pathogens, as they are more likely associated with rhabdomyolysis. Possible autoimmune etiologies, mainly idiopathic inflammatory myositis with possible CNS vasculitis involvement, were considered as well. Lastly, toxin mediated myopathy from metal poisoning was considered. Further workup included MRI of the brain and cervical spine which was unremarkable for vasculitis or encephalitis. Electromyogram and nerve conduction study showed a sensorimotor polyneuropathy with predominantly axonal features and moderate irritable myopathy. A lumbar puncture (LP) demonstrated 10 WBC/mm^3^ (80% lymphocytes), protein 50 mg/dL, normal glucose, and absence of oligoclonal bands. The lumbar puncture also demonstrated serologies positive for West Nile virus IgG and IgM antibodies but negative for EBV, HSV, VZV, CMV, VDRL, Lyme, toxoplasmosis, cysticercosis, AFB stain,* Cryptococcus*, coccidioidomycosis, bacterial cultures, fungal cultures, and viral cultures. Additional infectious disease workup included negative serologies for HIV screen, HIV RNA PCR, and HTLV I/II. Autoimmune workup demonstrated negative ANA, anti-Jo1, anti-Mi2, and anti-SRP. Other neurological workups performed included mercury levels and heavy metal levels that were undetectable.

The patient was subsequently diagnosed with WNV neuroinvasive disease, specifically West Nile poliomyelitis (WNP) and West Nile encephalitis (WNE) with myopathy/muscle involvement. With supportive care, the patient's CK and renal function trended back to normal over a period of five weeks. At the time of discharge, the patient had normal mental status, 4/5 strength in bilateral lower extremities requiring a cane for ambulation, and persistent right upper extremity weakness with 2/5 strength. At 6-month follow-up, he continued to have normal mental status, 4/5 strength in bilateral lower extremities requiring a cane for ambulation, and persistent right upper extremity weakness with 2/5 strength. At 12-month follow-up, his bilateral lower extremities improved to 5/5 strength and right upper extremity improved to 3/5 strength.

## 3. Discussion

West Nile virus (WNV) is a mosquito-borne RNA* Flavivirus* and human neuropathogen. During the early discovery of West Nile virus in the mid-1900s, common manifestations described included fevers, chills, malaise, maculopapular rash, headaches, backaches, arthralgia, and myalgia [4]. However, more recently neuromuscular manifestations are being recognized as a prominent feature in patients with WNV infection. The Centers for Disease Control (CDC) now classifies WNV infection into (1) WNV fever and (2) WNV neuroinvasive disease, with further subdivision of the latter group into (a) encephalitis, (b) meningitis, and (c) poliomyelitis. Patients with poliomyelitis commonly have signs of meningitis and encephalitis. Of all the people infected, 25% develop WNV fever [5] and neuroinvasive disease occurs in less than 1% [2]. Advancing age and immunosuppression were the risk factors associated with neuroinvasive disease [6].

More than 50% of patients with confirmed WNV encephalitis can have severe muscle weakness as a cardinal sign [7] which is considered a risk factor for predicting death in patients with WNV encephalitis [7, 8]. However, actual myopathy is an uncommon manifestation, with only several case reports of rhabdomyolysis with creatine kinase levels as high as 45,000 U/L [3, 9, 10]. Muscle biopsies of patients with acute asymmetric paralysis showed scattered necrotic muscle fibers invaded by macrophages [11]. The role of WNV directly invading the muscles remains unclear [4], but there have been case reports where staining with immunohistochemistry for polyclonal antibodies for* Flavivirus* was unremarkable [11]. A muscle biopsy was not pursued for our patient in this case, as our patient began to improve with symptomatic care alone. Additionally, as mentioned, the utility of a muscle biopsy for diagnosing WNV myopathy is still unclear given the rarity of this type of WNV manifestation.

West Nile virus can also cause myocarditis [12, 13] and cardiomyopathy [13, 14], commonly presenting as heart failure symptomology, cardiac arrhythmias, elevated cardiac enzymes, and new global myocardial dysfunction [12–14]. The diagnosis is usually made with the confirmation of WNV and with the exclusion of other etiologies, especially ischemic pathology [12–14]. In our patient, further diagnostics such as cardiac MRI were not pursued, as our patient did not have overt heart failure symptomology or new arrhythmias, and the troponin elevation resolved with symptomatic care. Additionally repeat echocardiogram imaging demonstrated improved ejection fraction compared to 5 months priorly and adenosine myoview showed fixed defect consistent with previous infarction. Our patient was thought to more likely have a non-ST elevation myocardial infarction from demand ischemia from acute illness, which he is at higher risk for given his past medical history of coronary artery disease and ischemic cardiomyopathy.

WNV infections should be considered especially during the summer in patients living in endemic areas presenting with unexplained acute illness or neurological findings. The incubation period ranges from 2 to 14 days. Serum should be tested for WNV IgM antibodies. If there is a neurological manifestation, then the cerebrospinal fluid (CSF) should be tested for WNV IgM antibodies. The CSF analysis will typically reveal increased leukocytes (usually >200 cells/mm^3^), increased protein, and normal glucose [4]. Imaging studies in WNV infection are frequently normal but are useful in eliminating other causes of acute meningoencephalitis. When abnormal, the findings are generally nonspecific. T2-weighted magnetic resonance signal abnormalities may be seen in brainstem, deep gray structures, cerebellum, and spinal cord ventral horns [15]. Other infectious entities that can present similarly to WNV should be ruled out as well, including HIV, CMV, EBV, HSV, VZV, HTLV1, Lyme, toxoplasmosis, cysticercosis, mycoplasma, and syphilis, with viral illnesses more likely to lead to rhabdomyolysis. Toxin mediated disease such as heavy metal poisoning should be considered if there is a relevant exposure history. Autoimmune etiologies such as idiopathic inflammatory myositis can be considered as well, but unless there is a concomitant CNS vasculitis, it is unlikely to lead to AMS [16].

The treatment is supportive and full recovery can be seen in uncomplicated cases of WNV fever or meningitis cases. Outcomes of WNV encephalitis are variable and patients can have substantial functional and cognitive difficulties for up to a year [2]. One-third of the patients with WNV poliomyelitis recover strength almost to baseline, one-third have minimal improvement, and one-third have no improvement [17]. Death among the patients with neuroinvasive disease is approximately 10% [6]. No vaccines are available. Mosquito control programs are the mainstay in prevention [2]. Finally, as treatment would be different, rheumatologists should be cognizant of the myopathic complications of WNV neuroinvasive disease as it may simulate an idiopathic inflammatory myopathy early on.
